# Molecular Landscape of *TP53*/*RB1* Co‐Altered Tumors Uncovers Emerging Therapeutic Vulnerabilities

**DOI:** 10.1002/gcc.70100

**Published:** 2026-01-21

**Authors:** Xuetao Li, Meifeng Ye, Xiaomei Huang, Zhong‐Yin Huang, Juan Zhu, Zipeng He, Zhuxiang Zhao, Jun Hou, Shuquan Wei

**Affiliations:** ^1^ Department of Pulmonary and Critical Care Medicine, Guangzhou First People's Hospital, The Second Affiliated Hospital South China University of Technology Guangzhou China; ^2^ Institute of Clinical Medicine, Guangzhou First People's Hospital, The Second Affiliated Hospital South China University of Technology Guangzhou China

**Keywords:** co‐alterations, pan‐cancer, *RB1*, therapeutic vulnerability, *TP53*

## Abstract

**Background:**

Although *TP53* and *RB1* co‐alterations play critical roles in promoting malignant development and progression, specific inhibitors targeting this co‐alteration are lacking. We performed a pan‐cancer analysis to characterize the biology of *TP53*/*RB1* co‐alterations and identify therapeutic strategies.

**Methods:**

We analyzed mutation data and copy number variation (CNV) data from 42 371 pan‐cancer samples across 26 cancer types from the cBioPortal database. Among them, 2417 tumors with *TP53*/*RB1* co‐alterations were used for further analysis. We characterized their epidemiology and molecular biology. Therapeutic vulnerabilities of co‐altered tumors were examined using Cancer Cell Line Encyclopedia drug screening datasets.

**Results:**

*TP53*/*RB1* co‐alterations occurred in 5.70% of pan‐cancer cases but exhibit striking heterogeneity across cancer types. Patients harboring co‐alterations had significantly shorter overall survival (OS) in both primary and metastatic settings and poorer response to immune checkpoint inhibitors. Co‐altered tumors displayed frequent alterations in chromatin remodeling genes (*CREBBP*, *ARID1A*, and *KMT2D*) and PI3K pathway (*PIK3CA* and *PTEN*), but with distinct tissue‐specific mutational patterns: *EGFR* mutations dominated in lung adenocarcinoma (52%), *KRAS* in pancreatic cancer (88%), and *APC* in colorectal cancer (77%). Moreover, upregulated genes in co‐altered tumors enriched in cell cycle pathways, DNA repair, and neuronal development, whereas immune/inflammatory signaling was suppressed. Critically, drug screening revealed that co‐altered tumors showed increased sensitivity to CDK, AURKA, and PI3K/mTOR inhibitors, but resistance to MAPK/ERK pathway inhibitors.

**Conclusions:**

*TP53*/*RB1* co‐alterations define an aggressive cancer subset with dysregulated cell cycle/chromatin pathways and reduced immunotherapy response. Targeting CDK, AURKA, or PI3K signaling offers promising therapeutic strategies.

## 1 Introduction

The tumor suppressor functions of *TP53* and *RB1* in preserving genomic stability and regulating cell cycle progression are well‐established across a wide spectrum of human malignancies. Genetic alterations in these genes critically promote malignant initiation and progression. In particular, *TP53* mutation (especially missense mutation) leads not only to loss of canonical tumor suppressive function, but may also confer gain‐of‐function properties that enhance tumor cell survival, invasiveness, and metastatic potential [1]. Similarly, *RB1* regulates the G1/S cell cycle checkpoint by inhibiting E2F transcription factors; its inactivation disrupts this regulatory axis, leading to uncontrolled cell cycle progression [2]. Importantly, *TP53*/*RB1* co‐alteration synergistically disrupts DNA damage response and cell cycle regulation, thereby fostering genomic instability, uncontrolled proliferation, and accelerating malignant transformation and tumor evolution [2, 3].

A comprehensive pan‐cancer study has revealed that *TP53*/*RB1* co‐alteration is the most prevalent co‐occurring genetic alteration across diverse cancer types, with particularly high frequencies in small‐cell carcinomas, neuroendocrine carcinomas, and sarcomas (SARCs) [4]. This co‐alteration is especially prevalent in small cell lung cancer (SCLC), where it occurs in approximately 59%–90% of cases and is regarded as a defining molecular event essential for SCLC pathogenesis [5, 6, 7]. Mechanistically, dual‐inactivation of *TP53* and *RB1* drives neuroendocrine transdifferentiation, contributing to the distinct morphological characteristics of SCLC [8]. Beyond SCLC, a systematic review revealed significant prevalence in other neuroendocrine cancers. Notably, *TP53*/*RB1* co‐alteration occurs in ~36% of pulmonary large cell neuroendocrine carcinoma (LCNEC) and ~35% of extra‐thoracic LCNEC patients [9]. Similarly, neuroendocrine prostate cancer exhibits a high incidence of this co‐alteration, with a reported prevalence of approximately 53.3% of cases [10].

Accumulating evidence indicates that tumors with concurrent loss of *TP53* and *RB1* exhibit a markedly increased propensity for transdifferentiating into small‐cell neuroendocrine tumor (SCNC). In EGFR‐mutant lung adenocarcinoma (LUAD), the combined inactivation of *TP53* and *RB1* significantly elevates the risk of small‐cell transformation following the development of resistance to EGFR tyrosine kinase inhibitors [3], while loss of *RB1* alone is insufficient to induce neuroendocrine differentiation [11]. Importantly, this transdifferentiation potential is not confined to lung cancer. Both prostate and urothelial cancers exhibit similar small‐cell transformation upon the loss of *TP53* and *RB1*. In prostate cancers, the dual loss of *TP53* and *RB1* promotes lineage plasticity in vitro and facilitates transdifferentiation to SCNC in vivo [12, 13], while in urothelial cancers, *TP53* and *RB1* loss occurring after the initial tumorigenesis is essential for the transdifferentiation into bladder small‐cell carcinoma [14]. Collectively, these findings across multiple cancer types strongly suggest that *TP53*/*RB1* co‐alteration is a convergent molecular program driving lineage plasticity and transdifferentiation to small‐cell neuroendocrine carcinoma in epithelial tumors.

Clinical evidence consistently links *TP53*/*RB1* co‐alterations to poor prognosis across key epithelial cancers, including EGFR‐mutant LUAD and prostate cancer [3, 15]. Given their adverse clinical implications, deciphering the molecular consequences of these co‐alterations is critical for developing targeted therapies aimed at improving outcomes in high‐risk patients. In this study, we conducted a comprehensive pan‐cancer analysis to delineate shared and cancer type‐specific molecular programs orchestrated by *TP53*/*RB1* co‐alterations across diverse malignancies and to identify actionable therapeutic vulnerabilities associated with these alterations.

## 2 Materials and Methods

### 2.1 Data Acquisition

This study utilized publicly available data. The mutation data and copy number variation data from 42 371 pan‐cancer samples across 26 cancer types were obtained from the cBioPortal (https://www.cbioportal.org/), which integrates datasets from the European Genome‐phenome Archive (EGA) (whole‐genome sequencing, WGS), The Cancer Genome Atlas (TCGA) (WGS or whole‐exome sequence, WES), and Memorial Sloan Kettering Cancer Center (MSKCC) (deep targeted sequencing). The paired transcriptome data from TCGA datasets (RNA‐seq). Mutation data of 1739 cancer cell lines and paired drug screening data of 1067 cancer cell lines were taken from the CCLE (https://portals.broadinstitute.org/ccle).

### 2.2 Identification of *TP53*/*RB1* Co‐Alterations

A somatic variant was removed if it met the following criteria: (1) synonymous or UTR variants; (2) the nonsilent mutations with < 2% mutation frequency. According to *TP53* and *RB1* alteration status (including point mutations, deletions, insertions, splice mutations, and copy number arm‐level losses), tumors were named as four genotypes: *TP53*/*RB1* co‐alteration (TP53/RB1‐CO), *RB1* alteration alone (RB1_only), *TP53* alteration alone (TP53_only), and *TP53*/*RB1* wildtype (TP53/RB1‐WT). The MutSigCV (v.1.41) [16] was applied to identify significantly mutated driver genes using a cutoff of false discovery rate < 0.1 for WGS/WES data. Finally, 2417 *TP53*/*RB1* co‐altered tumors were used for further analysis.

### 2.3 Differential Expression Analysis, Functional Enrichment Analysis, and Protein–Protein Interaction Network Analysis

We used DESeq2 to identify differentially expressed genes (DEGs) between TP53/RB1‐CO and non‐TP53/RB1‐CO tumors. The genes with |log2 fold change| > 1 and an adjusted *p* < 0.05 (Benjamini–Hochberg correction) were considered DEGs. Functional enrichment analysis was performed using Metascape [17] (http://metascape.org), a free web‐based portal that supports efficient data‐driven gene prioritization for multiple gene lists. Differentially expressed genes were uploaded to Metascape. Single‐sample gene set enrichment analysis (ssGSEA) was performed to determine enrichment scores for the 50 hallmark gene sets from the Molecular Signatures Database (MSigDB) using R package GSVA [18, 19]. The protein–protein interaction (PPI) network analysis was performed using the STRING database (https://string‐db.org/).

### 2.4 Statistical Analysis

Chi‐square testing was used for *p* value calculations across categorical variables, while the two‐sided Mann–Whitney *U* test was used across continuous variables. One‐way ANOVA was used to compare the differences in gene expression across four genotypes. Log rank test was used for comparison of survival distributions in Kaplan–Meier plots. Statistical analyses were conducted using R (version 4.5.0, https://www.r‐project.org/), SPSS (version 27.0, IBM, Armonk, NY, USA), and GraphPad Prism (version 9, GraphPad Software, La Jolla, CA, USA).

## 3 Results

### 3.1 *TP53*/*RB1* Co‐Alterations Across Various Tumors

**FIGURE 1:**
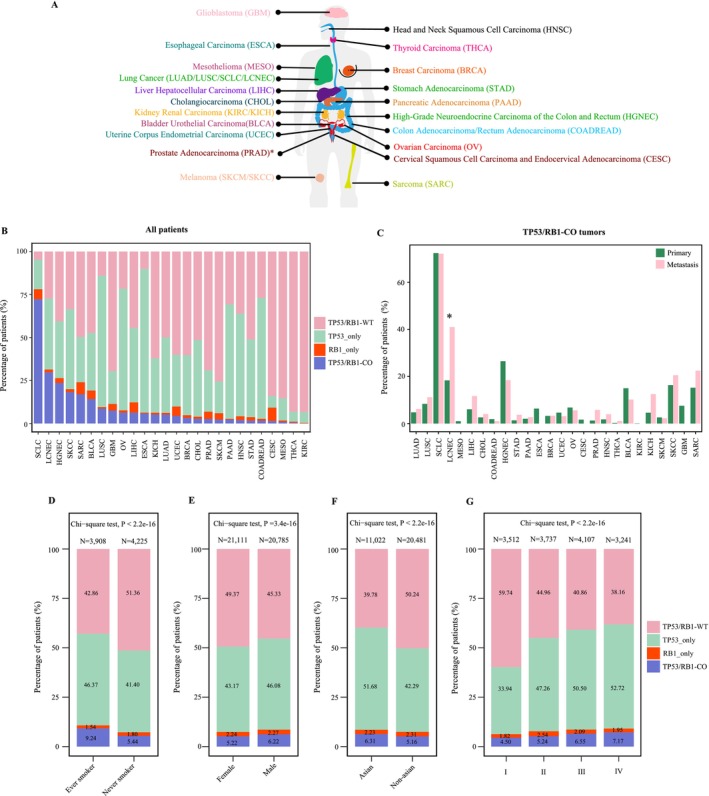
The distribution of different *TP53*/*RB1* alteration status in pan‐cancer. (A) Schematic overview of the pan‐cancer in our cohort. (B) The proportional distribution of four *TP53*/*RB1* alteration genotypes across 26 cancer types. (C) The proportional distribution of *TP53*/*RB1* co‐alteration between primary and metastasis samples. (D–G) The proportional distribution of different *TP53*/*RB1* alteration status (D) in ever‐smokers and never‐smokers, (E) in females and males, (F) in Asian and non‐Asian patients, and (G) across tumor stages. *p* values were calculated by chi‐square test.

To determine the frequency of *TP53*/*RB1* co‐alterations across various tumors, we analyzed somatic mutation data and copy number variation data from 42 371 pan‐cancer samples spanning 26 distinct cancer types, covering malignancies of the respiratory, digestive, reproductive, and urinary systems, among others (Figure 1A; Table 1). *TP53*/*RB1* co‐alterations were identified in 2417 out of the 42 371 pan‐cancer samples (5.70%). The prevalence of these co‐alterations varied significantly across different cancer types. A particularly high frequency of *TP53*/*RB1* co‐alteration was observed in respiratory system tumors (964/8835 cases, 10.91%), with the highest frequency observed in SCLC (475/657 cases, 72.30%), followed by LCNEC (36/121 cases, 29.75%), lung squamous cell carcinoma (LUSC) (123/1411 cases, 8.72%), and LUAD (327/6312 cases, 5.18%). In urinary system tumors, the frequency of co‐alteration was 14.22% in bladder urothelial (BLCA), 5.26% in chromophobe renal cell carcinoma (KICH), and 0.08% in clear cell renal cell carcinoma (KIRC). Notably, elevated co‐alteration rates were also found in other neuroendocrine carcinomas besides LCNEC, such as high‐grade neuroendocrine carcinoma (HGNEC) (23.58%). Additional tumor types exhibiting relatively high *TP53*/*RB1* co‐alteration frequencies included skin cutaneous carcinoma (SKCC) (18.24%), SARC (16.99%), glioblastoma multiforme (GBM) (7.54%), liver hepatocellular carcinoma (LIHC) (6.30%), and esophageal carcinoma (ESCA) (5.81%) (Table 1).

**TABLE 1:** The distribution of *TP53*/*RB1* co‐alterations across different systems within the studied sample population.

System	Organ	Histological subtypes	Total samples	*TP53*/*RB1* co‐alterations	Co‐alteration percentage (%)
Respiratory			8835	964	10.91
	Lung	LUAD	6312	327	5.18
		LUSC	1411	123	8.72
		SCLC	657	475	72.30
		LCNEC	121	36	29.75
	Pleural	MESO	334	3	0.90
Digestive			13 987	404	2.89
	Liver	LIHC	1667	105	6.30
		CHOL	979	27	2.76
	Colorectal	COADREAD	5443	89	1.64
		HGNEC	106	25	23.58
	Stomach	STAD	2029	34	1.68
	Pancreas	PAAD	2662	60	2.25
	Esophagus	ESCA	1101	64	5.81
Reproductive			11 129	421	3.78
	Breast	BRCA	4213	137	3.25
	Uterus	UCEC	1801	79	4.39
	Ovary	OV	2026	128	6.32
	Cervical	CESC	400	6	1.50
	Prostate	PRAD	2689	71	2.64
Head and neck			1781	20	1.12
	Head and neck	HNSC	759	15	1.98
	Thyroid	THCA	1022	5	0.49
Urinary			3018	246	8.15
	Bladder	BLCA	1688	240	14.22
	Kidney	KIRC	1235	1	0.08
		KICH	95	5	5.26
Skin	Skin		1337	58	4.34
		SKCM	1167	27	2.31
		SKCC	170	31	18.24
Nervous	Brain	GBM	889	67	7.54
Soft Tissue	Soft tissue	SARC	1395	237	16.99
Total			42 371	2417	5.70

Abbreviations: BLCA, bladder carcinoma; BRCA, breast carcinoma; CESC, cervical squamous cell carcinoma and endocervical adenocarcinoma; CHOL, cholangiocarcinoma; COADREAD, colon adenocarcinoma/rectum adenocarcinoma; ESCA, esophageal carcinoma; GBM, glioblastoma multiforme; HGNEC, high‐grade neuroendocrine carcinoma of the colon and rectum; HNSC, head and neck squamous cell carcinoma; KICH, kidney chromophobe carcinoma; KIRC, kidney renal clear cell carcinoma; LCNEC, pulmonary large cell neuroendocrine carcinoma; LIHC, liver hepatocellular carcinoma; LUAD, lung adenocarcinoma; LUSC, lung squamous cell carcinoma; MESO, pleural mesothelioma; OV, ovarian cancer; PAAD, pancreatic adenocarcinoma; PRAD, prostate adenocarcinoma; SARC, sarcoma; SKCC, skin cancer, nonmelanoma; SKCM, skin cutaneous melanoma; STAD, stomach adenocarcinoma; THCA, thyroid carcinoma; UCEC, uterine corpus endometrial carcinoma.

We next investigated the proportional distribution of *TP53*/*RB1* alteration status across 26 cancer types. In most cancer types, the number of samples harboring *TP53*/*RB1* co‐alterations was substantially higher than those with *RB1* alterations alone, whereas *TP53‐*only alterations were prevalent overall (Figure 1B). Across most cancer types, the frequency of *TP53*/*RB1* co‐alterations was comparable between primary and metastatic tumors (Figure 1C). In contrast, a significantly elevated frequency was observed in metastatic versus primary LCNEC samples (*p* = 0.0004).

When assessing the relationship between *TP53*/*RB1* alteration status and clinicopathological characteristics, a significantly higher frequency of co‐alterations was observed in smokers compared to nonsmokers (9.24% vs. 5.44%, Figure 1D). In contrast, sex and race showed only weak associations with *TP53*/*RB1* co‐alteration status: males versus females (6.22% vs. 5.22%) and Asians versus non‐Asians (6.31% vs. 5.16%) (Figure 1E,F). Notably, the frequencies of *TP53*/*RB1* co‐alterations and *TP53‐*only alterations were higher in tumors of advanced stages (Stages III and IV), whereas TP53/RB1‐WT frequencies were significantly higher in early‐stage tumors (Stage I) compared to Stages II, III, and IV (Figure 1G).

### 3.2 *TP53*/*RB1* Co‐Alteration Is Associated With Poor Clinical Outcomes

**FIGURE 2:**
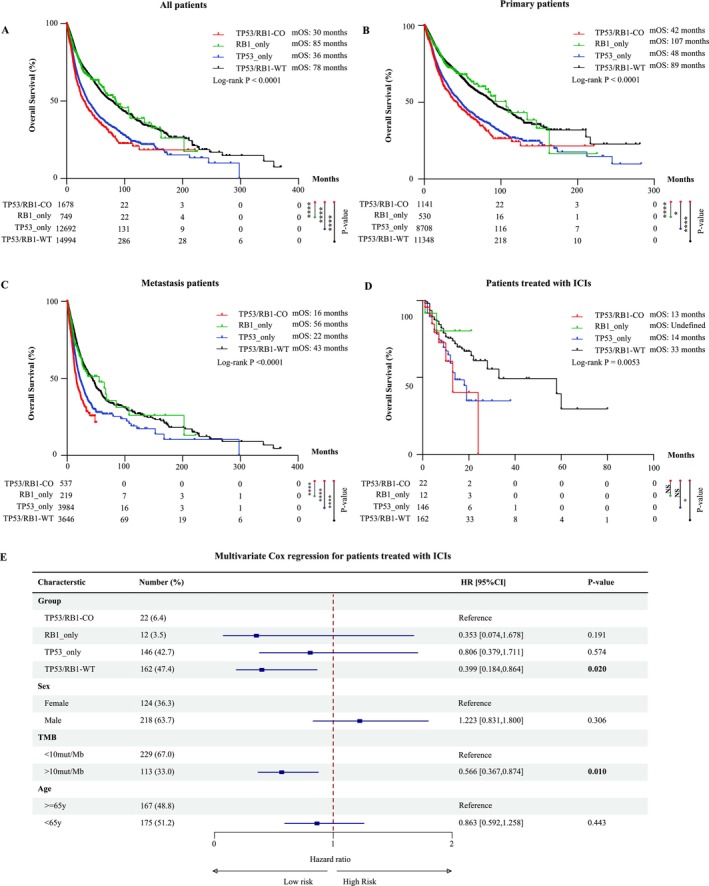
The effect of different *TP53*/*RB1* alteration status on OS. (A) Kaplan–Meier curves for OS in all patients, stratified by *TP53*/*RB1* alteration status. The log rank test (B, C) Kaplan–Meier curves for OS in primary patients (B) and metastasis patients (C). (D) Kaplan–Meier curves for OS in patients treated with ICIs. (E) Table and forest plot showing the results of multivariate COX regression analysis of *TP53*/*RB1* alteration status and clinicopathological variables in patients treated with ICIs. Hazard ratios are presented with their 95% confidence interval. *p* values of statistical significance are represented as *p < 0.05, **p < 0.01, ***p < 0.001, and ****p < 0.0001.

To explore the prognostic significance of concurrent *TP53/RB1* alterations, we investigated the correlation between four genotypic subgroups (based on *TP53* and *RB1* mutation status) and the prognosis of patients across a pan‐cancer cohort. Patients with co‐alterations had statistically significant shorter median OS (mOS) compared to all other groups in pan‐cancer patients (Figure 2A). This adverse prognostic effect persisted when stratifying patients by sample type (primary cohort vs. metastasis cohort), where co‐altered cases consistently demonstrated inferior mOS in both primary and metastatic samples (Figure 2B,C). Notably, patients with *TP53*/*RB1*‐WT or *RB1‐*only alterations exhibited markedly longer mOS compared to those with co‐alterations or *TP53*‐only alterations. These data suggest that *TP53*/*RB1* co‐alteration may serve as a poor prognostic marker across diverse tumor types.

We further assessed the impact of *TP53* and *RB1* alteration status on clinical response to immune checkpoint inhibitors (ICIs). Surprisingly, it was found that patients with co‐alterations had the shortest mOS (13 months) (Figure 2D), despite the fact that patients with co‐alterations harbor an intermediate to high tumor mutation burden (TMB) (Figure S1). Conversely, patients with *TP53*/*RB1*‐WT demonstrated the longest mOS (33 months). Due to the limited number of patients with *RB1*‐only alterations (*n* = 10), mOS could not be estimated for this group. Multivariate Cox regression analysis demonstrated that the *TP53* and *RB1* alteration status may independently predict clinical response to ICIs, alongside previously established markers such as TMB (Figure 2E).

### 3.3 Tissue‐Specific Mutational Landscapes in *TP53*/*RB1* Co‐Altered Tumors

**FIGURE 3:**
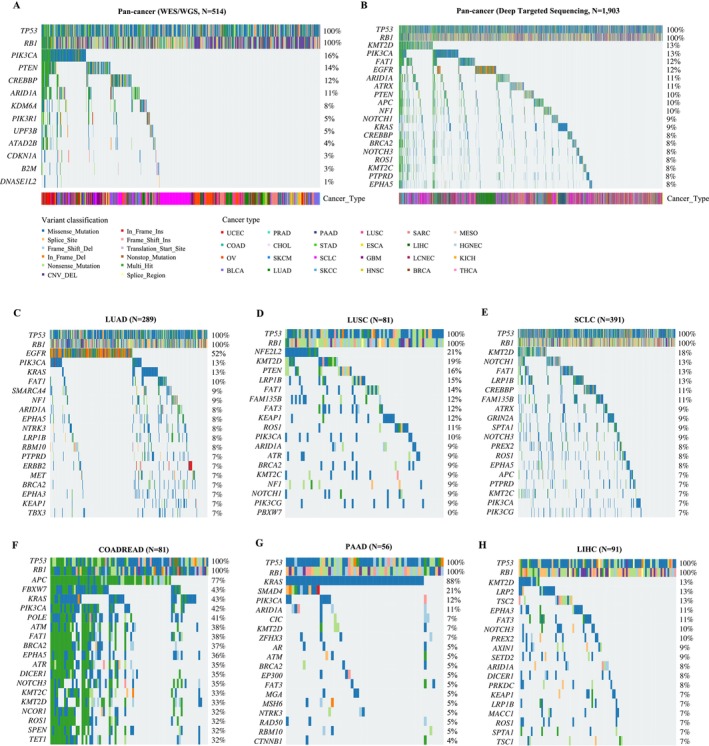
Mutational landscape of *TP53*/*RB1* co‐altered tumors. (A, B) Pan‐cancer landscape detected by (A) WES/WGS and (B) deep targeted sequencing, respectively. (C–G) Mutational landscape in specific tumor types: (C) LUADs, (D) LUSC, (E) SCLC, (F) COADREAD, (H) PAAD, and (G) LIHC.

To characterize the mutational landscapes of *TP53*/*RB1* co‐altered tumors, we investigated somatic mutations and copy number variation using WES/WGS, targeted deep sequencing datasets, and copy number variation arrays. With pooling gene alterations across 514 patients with *TP53*/*RB1* co‐alterations in the WES/WGS cohort, we identified 11 driver mutations besides *TP53* and *RB1*. Of these, the most frequently co‐altered genes were *PIK3CA* (16%), *PTEN* (14%), *CREBBP* (12%), and *ARID1A* (11%) (Figure 3A). A similar mutational profile was observed in the targeted deep sequencing dataset of *TP53*/*RB1* co‐altered tumors, with frequent co‐alterations in *PIK3CA* (13%), *PTEN* (10%), *CREBBP* (8%), and *ARID1A* (11%) (Figure 3B). It is important to note that these recurrent co‐occurring alterations predominantly involve genes implicated in chromatin remodeling (*CREBBP*, *ARID1A*) and the PI3K signaling pathway (*PIK3CA*, *PTEN*). In addition, frequently co‐altered genes in *TP53*/*RB1* co‐altered tumors included *KMT2D* (13%), *FAT1* (12%), *EGFR* (12%), *ATRX* (11%), and *APC* (10%).

Next, we investigated tissue‐specific mutational landscapes in *TP53*/*RB1* co‐altered tumors of various cancer types. *EGFR* mutations were identified in nearly 52% of *TP53*/*RB1* co‐altered LUAD (Figure 3C), suggesting that *EGFR*/*TP53*/*RB1* triple‐mutant tumors may define a highly plastic subset with potential for SCLC transdifferentiate [20]. Other frequently mutated genes in *TP53*/*RB1*‐mutant LUADs included *PIK3CA* (13%) and *KRAS* (13%). It is important to note that *TP53*/*RB1* co‐altered LUSC and SCLC exhibited similar frequencies of genomic alterations in *KMT2D*, *FAT1*, and *LRP1B* (Figure 3D,E). However, *NFE2L2* (21%) and *PTEN* (16%) mutations were more frequent in LUSC, whereas *NOTCH1* (13%) and *CREBBP* (11%) alterations were more common in SCLC.

Besides thoracic malignancies, distinct co‐alteration patterns were also observed in gastrointestinal tumors. In *TP53*/*RB1* co‐mutant colon adenocarcinoma and rectum adenocarcinoma (COADREAD), highly recurrent genomic alterations were identified in a core set of genes, including *APC* (77%), *FBXW7* (43%), *KRAS* (43%), *PIK3CA* (42%), and *POLE* (41%) (Figure 3F). In *TP53*/*RB1* co‐altered pancreatic adenocarcinoma (PAAD), *KRAS* mutations were nearly ubiquitous (88%), and a notable rate of mutations was noticed in *SMAD4* (21%) (Figure 3G). In hepatocellular carcinoma (LIHC), *TP53*/*RB1* co‐altered tumors frequently harbored concurrent *KMT2D* (13%) and *LRP2* (13%) alterations (Figure 3H). Tissue‐specific mutational landscapes were also observed in other cancer types (Figure S2).

### 3.4 *TP53*/*RB1* Co‐Altered Tumors Exhibit Distinct Biological Pathways

**FIGURE 4:**
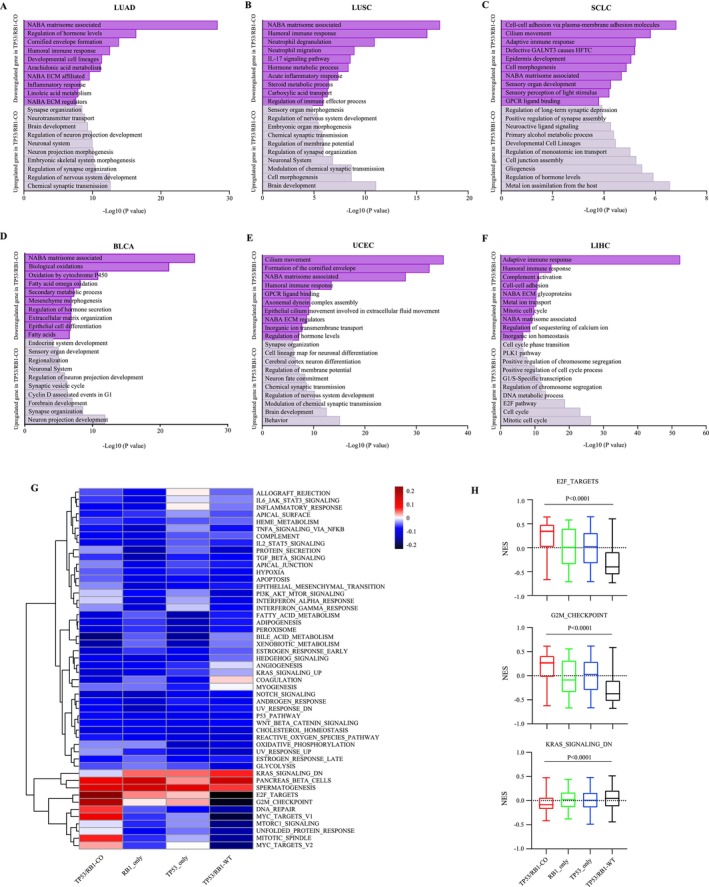
Biological pathways in *TP53*/*RB1* co‐altered tumors. (A–F) Pathway enrichment analysis for up‐ and downregulated genes in *TP53*/*RB1* co‐altered tumors compared with non‐co‐altered tumors based on KEGG, Reactome, and GO gene sets. Significantly enriched pathways in (A) LUAD, (B) LUSC, (C) SCLC, (D) BLCA, (E) UCEC, and (F) LIHC. (G) Heatmap of GSEA normalized enrichment score (NES) for hallmark gene sets among four *TP53*/*RB1* alteration genotypes. (H) Comparison of NES for selected gene sets (E2F_targets, G2M_checkpoint, and KRAS_signaling_dn) among four *TP53*/*RB1* alteration genotypes. *p* values were calculated by one‐way ANOVA.

To identify the biological differences between the *TP53*/*RB1* co‐altered and non‐co‐altered tumors, we performed gene set enrichment analyses based on DEGs. Across the majority of tumor types, *TP53*/*RB1* co‐altered tumors were significantly enriched for gene sets related to neuronal development and cell cycle progression, whereas those related to extracellular matrix (NABA matrisome associated) and to immune response and inflammation were consistently de‐enriched gene sets (Figure 4A–F and Figure S3).

Next, we performed ssGSEA using the 50 hallmark gene sets based on total gene expression to investigate the underlying biological features of the four genotypes. Similarly, gene sets related to E2F_targets, G2M_checkpoint, DNA_repair, MYC_targets, and MITOTIC_spindle were significantly enriched in *TP53*/*RB1* co‐altered tumors (Figure 4G,H). These results suggest that biological processes governing cancer cell behavior differ substantially across different *TP53*/*RB1* genotypes and that *TP53*/*RB1* co‐altered tumors tend to activate specific sets of oncogenic pathways across cancer types.

### 3.5 *TP53*/*RB1* Co‐Altered Tumors Possess Potential Therapeutic Vulnerabilities

**FIGURE 5:**
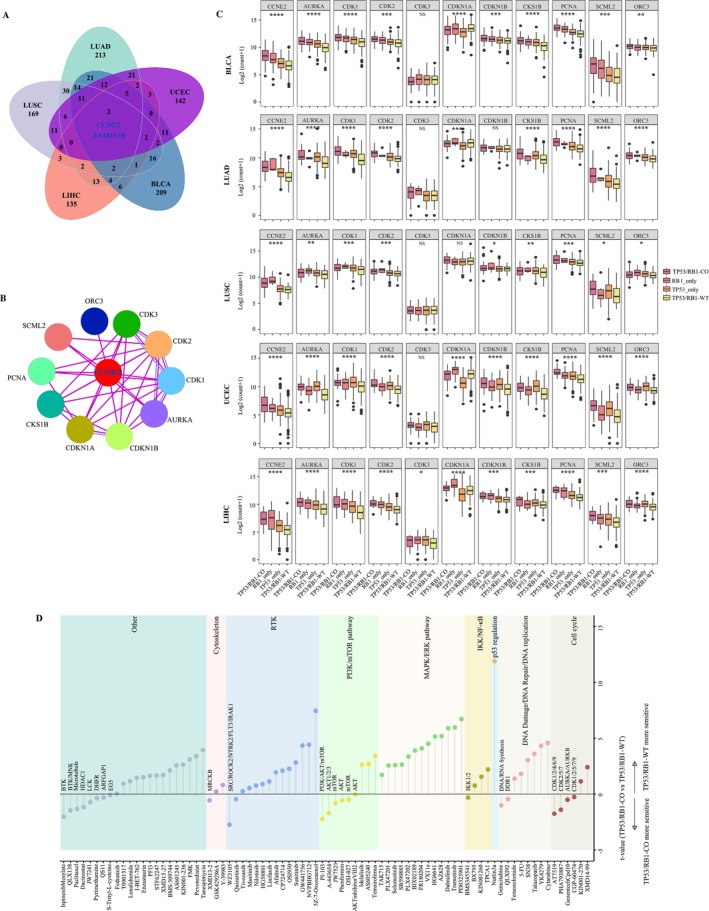
Potential therapeutic vulnerabilities of *TP53*/*RB1* co‐altered tumors. (A) Venn diagram of overlapping genes across *TP53*/*RB1* co‐altered tumors from LUAD, LUSC, BLCA, UCEC, and LIHC. (B) Protein–protein interaction network highlighting CCNE2 and its 10 interactors. (C) Expression levels of CCNE2 and its interactors across four *TP53*/*RB1* alteration genotypes from LUAD, LUSC, BLCA, UCEC, and LIHC. (D) Differential drug sensitivity between *TP53*/*RB1*‐CO and *TP53*/*RB1*‐WT cancer cell lines. The *t* value from *t*‐test of IC50 values of *TP53*/*RB1*‐CO versus *TP53*/*RB1*‐WT cancer cell lines. *p* values of statistical significance are represented as *p < 0.05, **p < 0.01, ***p < 0.001, and ****p < 0.0001.

Given the poor prognosis associated with *TP53*/*RB1* co‐altered tumors, identifying novel genotype‐specific therapeutic targets is of high clinical relevance. We first identified commonly upregulated genes in *TP53*/*RB1* co‐altered tumors across LUAD, LUSC, BLCA, UCEC, and LIHC, revealing CCNE2 and FAM111B as consistently overexpressed candidates (Figure 5A). CCNE2, a critical regulator of the G1/S cell cycle transition and an oncogene implicated in multiple cancers [21], was prioritized for further analyses. To explore its druggable potential, we constructed a PPI network using the STRING database, identifying 10 direct interactors: *AURKA*, *CDK1*, *CDK2*, *CDK3*, *CDKN1A*, *CDKN1B*, *CKS1B*, *PCNA*, *SCML2*, and *ORC3* (Figure 5B). Notably, most interactors exhibited significantly elevated expression in *TP53*/*RB1* co‐altered tumors compared to other genotypes (Figure 5C), highlighting a coordinated activation of cell cycle‐related proteins.

To investigate potential therapeutic vulnerabilities of *TP53*/*RB1* co‐altered tumors, we leveraged CCLE drug screening datasets comprising 266 compounds. This analysis identified 80 compounds with differential efficacy between co‐altered and *TP53*/*RB1*‐WT tumors (Figure 5D; Table S1). Co‐altered tumors showed increased sensitivity to cell cycle inhibitors, including CDK inhibitors (AT7519, PHA793887, CGP60474), AURKA/AURKB inhibitor (GenentechCpd10), as well as agents targeting the PI3K/mTOR pathway (Figure 5D). In contrast, co‐altered tumors exhibited resistance to receptor tyrosine kinase (RTK) inhibitors and MAPK/ERK pathway inhibitors, such as Trametinib and Dabrafenib (Figure 5D). Collectively, these findings highlight the unique therapeutic vulnerabilities of *TP53*/*RB1* co‐altered tumors and suggest that targeting cell cycle and PI3K/mTOR signaling pathways may represent effective treatment strategies in this high‐risk cancer subgroup.

## 4 Discussion

*TP53*/*RB1* co‐alterations are recurrently detected across a wide spectrum of cancer types and act synergistically to promote unrestricted proliferation, loss of G1/S checkpoint controls, and replication stress. Although direct therapeutic targeting of *TP53* and *RB1* remains challenging due to the nature of their loss‐of‐function mutations, interventions aimed at mitigating their downstream effects hold considerable therapeutic promise. In this study, we performed a comprehensive pan‐cancer analysis to elucidate the molecular and clinical impact of *TP53*/*RB1* co‐aberrations, paving the foundation for developing novel therapeutic strategies.

This large‐scale pan‐cancer analysis reveals that *TP53*/*RB1* co‐alterations occur in approximately 5.70% of pan‐cancer, though exhibiting striking heterogeneity across histology. The prevalence of these co‐alterations is exceptionally high in SCLC and other neuroendocrine carcinomas (e.g., LCNEC and HGNEC). Beyond neuroendocrine tumors, significantly elevated frequencies were also observed in SKCC, SARC, BLCA, and GBM, suggesting these co‐alterations as a potential oncogenic driver in diverse contexts. Interestingly, elevated co‐alteration rates in metastatic LCNEC imply that these alterations may promote metastatic fitness.

Of particular relevance is the impact of *TP53*/*RB1* co‐alteration on clinical outcomes. Consistent with prior reports, *TP53*/*RB1* co‐alterations are consistently associated with poor clinical outcomes across solid tumors [2, 4, 22]. Importantly, our results confirm that this adverse prognostic association persists in both primary and metastatic disease, underscoring their driving role to confer aggressive biological behavior independent of stage. Notably, the adverse association of these co‐alterations with OS is recalled in patients receiving immunotherapy, despite exhibiting intermediate‐to‐high TMB. We hypothesize that this impaired immunotherapy response may stem from an immunosuppressive tumor microenvironment, as previous research has linked loss of *TP53* and *RB1* with diminished immune infiltration [23]. Nevertheless, further mechanistic studies are required to elucidate how *TP53*/*RB1* co‐alterations drive immune evasion and resistance to immunotherapy.

Our comprehensive genomic profiling reveals a convergent mutational landscape in *TP53*/*RB1* co‐altered tumors, characterized by frequent alterations in chromatin remodeling and PI3K signaling pathways, suggesting *TP53*/*RB1* loss drives tumor progression through broader oncogenic programs beyond core cell cycle dysregulation [24]. The dual‐inactivation of *TP53* and *RB1* initiates epigenetic reprogramming events through upregulation of key regulators such as EZH2 and SOX2, fostering a stem cell‐like epigenetic state that ultimately drives tumor development and progression [12]. Notably, tissue‐specific mutational patterns were observed in *TP53*/*RB1* co‐altered tumors, demonstrating their biological diversity. For instance, most *TP53*/*RB1* co‐altered LUADs harbor EGFR mutations as well. Consistent with previous studies, LUAD tumors harboring *EGFR*/*TP53*/*RB1* co‐alterations are more likely to undergo SCLC transdifferentiation into SCLC [22], a phenotypic switch contributing to acquired resistance to EGFR inhibitors [25]. Further studies are needed to better elucidate the molecular mechanisms underlying *EGFR*/*TP53*/*RB1* co‐alterations in modulating neuroendocrine differentiation and lineage plasticity.

*TP53*/*RB1* co‐altered SCLCs are enriched with co‐occurring alterations in NOTCH1 (neuroendocrine suppressor) and *CREBBP* (epigenetic regulator), in line with their neuroendocrine identity [26, 27]. *TP53*/*RB1* co‐altered COADREADs display high mutation rates in components of the Wnt (*APC*) and Ras (*KRAS* and *FBXW7*) signaling pathways. Similarly, *TP53*/*RB1* co‐altered PAADs are nearly ubiquitously driven by *KRAS* mutation, with a notable subset also harboring *SMAD4* mutations, implicating TGF‐β pathway disruption. Across multiple tumor types, *TP53* and *RB1* co‐alteration upregulates pathways involved in the cell cycle, DNA damage repair, and neuron development and function. The enrichment of neuronal development and function pathways may directly reflect the activation of the transcriptional program driving neuroendocrine transdifferentiation. However, significant upregulation of neuronal development and function pathways was not observed in *TP53*/*RB1* co‐altered neuroendocrine tumors (such as SCLC). A possible explanation is that the intrinsic neuroendocrine properties in these tumors are not solely dictated by *TP53* and *RB1* inactivation. Although these mutations are characteristic of neuroendocrine tumors, particularly those with small cell or large cell histology, they do not fully account for the neuroendocrine phenotype. Functional validation studies are needed to establish causality between *TP53*/*RB1* co‐alterations and neuronal pathway activation.

While no current therapies directly target *TP53* and *RB1* co‐alterations, targeting their downstream effectors is a promising therapeutic strategy—particularly DNA repair and cell cycle regulators [28]. Preclinical evidence suggests that combined inhibition of PARP and ATR significantly suppresses the growth of *TP53*/*RB1* co‐altered prostate cancer cells [2]. In addition, CHK1 inhibitors may be effective, as *TP53*/*RB1* loss leads to compensatory upregulation of the ATR/CHK1 pathway in preclinical studies, which tumor cells depend on to enforce the G2/M checkpoint for DNA damage repair [29].

In this study, we identified CCNE2 as a gene consistently upregulated across multiple *TP53*/*RB1* co‐altered tumors, including LUAD, LUSC, BLCA, LIHC, and UCEC. CCNE2 plays a crucial role in regulating the cell cycle, specifically at the G1/S transition [30], and its dysregulation can drive uncontrolled proliferation and oncogenesis [31]. Overexpression of CCNE2 directly induces hyperactivation of CDK2, and their interactions promote the formation of the CCNE2/CDK2 complex, which promotes the G1/S transition of the cell cycle [21]. Although no specific inhibitors of CCNE2 currently exist, CDK2 inhibitors have shown efficacy in suppressing the proliferation of CCNE2‐overexpressing breast cancer cells. The upregulation of both CCNE2 and CDK2 in *TP53*/*RB1* co‐mutant tumors suggests that the CCNE2–CDK2 axis is a therapeutically targetable vulnerability, as corroborated by drug screening from the CCLE (Figure 5D). In addition, *TP53*/*RB1* co‐altered tumors showed increased sensitivity to AURKA inhibitors, potentially attributable to high AURKA expression. It is important to note that *TP53*/*RB1* co‐altered tumors also showed heightened sensitivity to PI3K/mTOR inhibition, consistent with a genomic landscape enriched for alterations in the PI3K signaling pathway.

Our study has several limitations. First, the analyzed data were retrieved from multiple publicly available datasets. Due to variations in sequencing platforms (WES/WGS, targeted panels), depth of coverage, and bioinformatic pipelines for mutation calling may introduce bias in mutation detection of *TP53* and *RB1*. Second, although *TP53*/*RB1* co‐inactivation is a hallmark of neuroendocrine tumorigenesis, it is also frequently detected in non‐neuroendocrine tumors. Due to the limited number of neuroendocrine tumor samples in our cohort, we were unable to conduct a comparative analysis of the effects of *TP53*/*RB1* co‐alteration in neuroendocrine versus non‐neuroendocrine tumors. Third, due to incomplete clinical information, the multivariate Cox model for evaluating ICI response only included a limited set of variables, including TMB, *TP53*/*RB1* alteration status, sex, and age. Additional factors such as PD‐L1 expression, prior therapies, and immune cell infiltration, which may influence ICI response, were not accounted for. Future clinical studies in larger, well‐annotated patient cohorts will be essential to validate and expand upon these findings.

## Author Contributions

Study concept and design: Xuetao Li, Jun Hou, and Shuquan Wei. Analysis and interpretation of data: Xuetao Li. Statistical analysis: Xuetao Li and Meifeng Ye. Drafting of the manuscript: Xuetao Li, Jun Hou, and Shuquan Wei. Critical revision of the manuscript for important intellectual content: Zhuxiang Zhao and Zhong‐Yin Huang. Obtained funding: Shuquan Wei. Study supervision: Jun Hou and Shuquan Wei. All authors had full access to the data in the study and take responsibility for the integrity of the data and the accuracy of the data analysis. All authors read and approved the final manuscript.

## Funding

This work was supported by Science and Technology Program in Guangzhou (Grant number 2024A03J0960) and National Natural Science Foundation of China (Grant Number 82400118).

## Ethics Statement

The authors have nothing to report.

## Conflicts of Interest

The authors declare no conflicts of interest.

## Supporting information


**Figure S1:** Comparison of TMB across four *TP53*/*RB1* mutation genotypes.
**Figure S2:** Mutational landscape in specific tumor types: (A) Respiratory system tumor (LCNEC); (B) Digestive system tumors (ESCA, CHOL, HGNEC); (C) Reproductive system tumors (BRCA, UCEC, OV, PRAD); (D) Urinary system tumors (BLCA); (E) GBM; (F) SARC.
**Figure S3:** Pathway enrichment analysis for up‐ and downregulated genes in *TP53*/*RB1* co‐mutated tumors compared with non‐non‐co‐mutated tumors based on KEGG, Reactome, and GO gene sets. Significantly enriched pathways in (A) GBM, (B) SARC.

**Table S1:** Eighty compounds were found to exhibit differential efficacy between TP53/RB1‐CO and TP53/RB1‐WT tumors.

## Data Availability

Publicly available datasets were analyzed in this study. Human pan‐cancer data can be found from the cBioPortal (https://www.cbioportal.org/). Pan‐cancer cell lines data can be found from the Cancer Cell Line Encyclopedia (https://portals.broadinstitute.org/ccle).
