# Supplementary material for: Molecular Landscape of TP53/RB1 Co‐Altered Tumors Uncovers Emerging Therapeutic Vulnerabilities
Source: Genes Chromosomes Cancer. 2026 Jan 21;65(1):e70100. doi: 10.1002/gcc.70100 (PMC12820914; doi:10.1002/gcc.70100)
Supplement: Supplementary file 1 — Figure S1: Comparison of TMB across four TP53/RB1 mutation genotypes. Figure S2: Mutational landscape in specific tumor types: (A) Respiratory system tumor (LCNEC); (B) Digestive system tumors (ESCA, CHOL, HGNEC); (C) Reproductive system tumors (BRCA, UCEC, OV, PRAD); (D) Urinary system tumors (BLCA); (E) GBM; (F) SARC. Figure S3: Pathway enrichment analysis for up‐ and downregulated genes in TP53/RB1 co‐mutated tumors compared with non‐non‐co‐mutated tumors based on KEGG, Reactome, and GO gene sets. Significantly enriched pathways in (A) GBM, (B) SARC. [file GCC-65-e70100-s001.pdf]

Figure S1

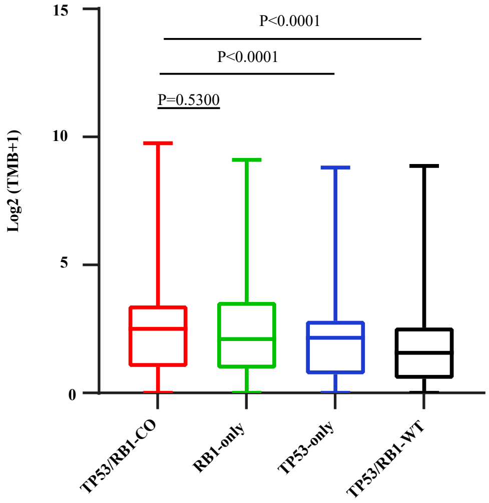

Figure S1 Comparison of TMB across four *TP53*/*RB1* mutation genotypes

Figure S2

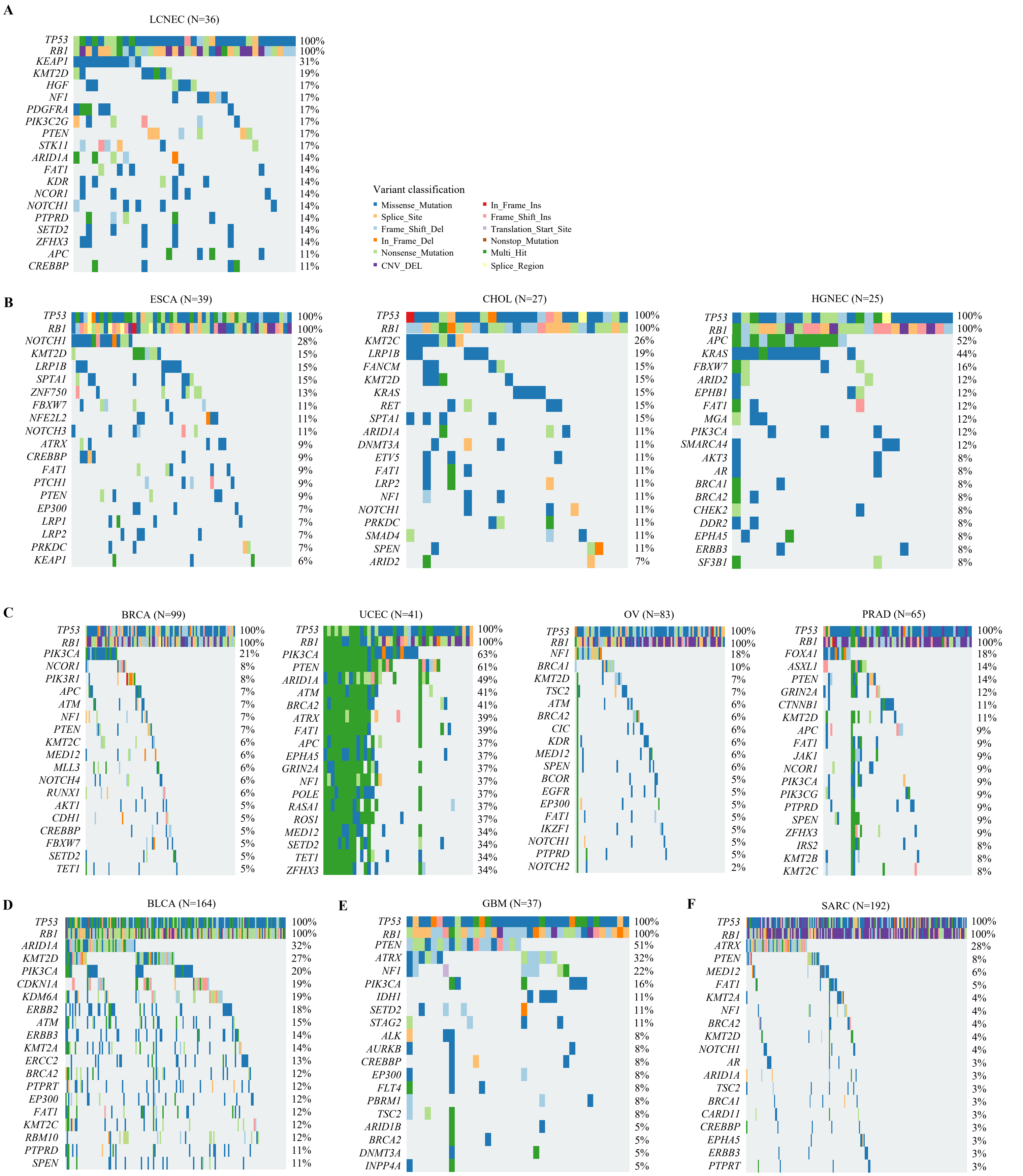

Figure S2 Mutational landscape in specific tumor types: (**A**) Respiratory system tumor (LCNEC); (**B**) Digestive system tumors (ESCA, CHOL, HGNEC); (**C**) Reproductive system tumors (BRCA, UCEC, OV, PRAD); (**D**) Urinary system tumors (BLCA); (**E**) GBM; (**F**) SARC.

Figure S3

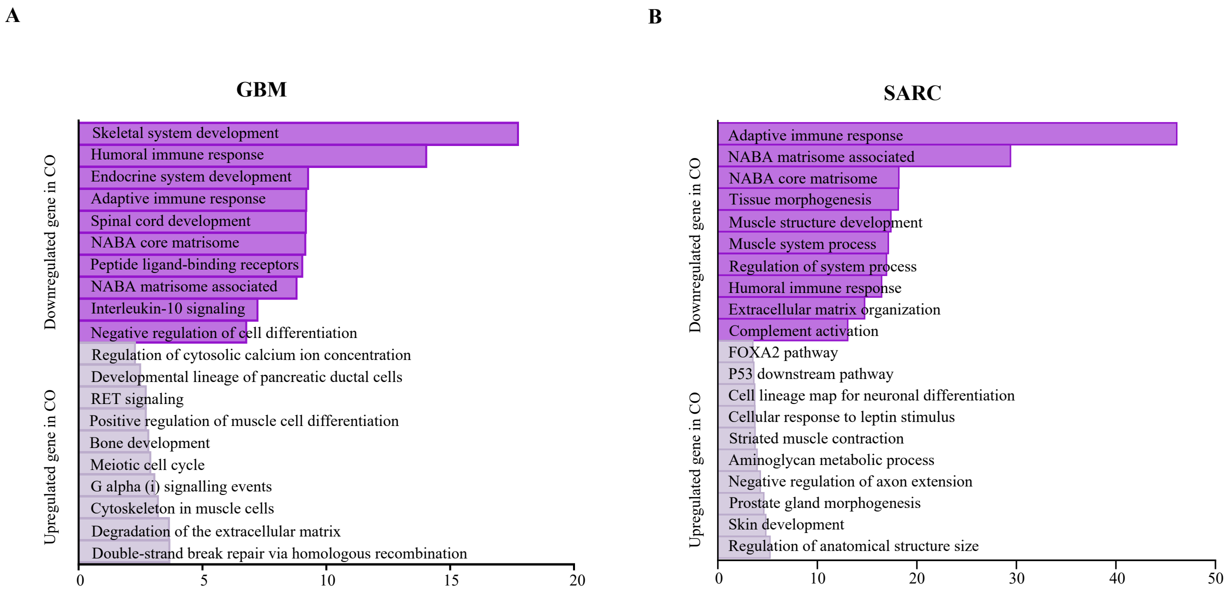

Figure S3 (A-B) Pathway enrichment analysis for up- and down-regulated genes in *TP53*/*RB1* co-mutated tumors compared with non-non-co-mutated tumors based on KEGG, Reactome, and GO gene sets. Significantly enriched pathways in (**A**) GBM, (**B**) SARC.
